# Tele-Intensive Care Unit Program in Brazil: Implementation and Expansion

**DOI:** 10.1089/tmr.2023.0017

**Published:** 2023-05-25

**Authors:** Paula Gobi Scudeller, Celina de Almeida Lamas, Aline Morgan Alvarenga, Michelle Louvaes Garcia, Talita Freitas Amaral, Martina Rodrigues de Oliveira, Bruno Rocha de Macedo, Carolina Burgarelli Testa, Fernanda Spadotto Baptista, Rossana Pulcineli Vieira Francisco, Carlos Roberto Ribeiro de Carvalho

**Affiliations:** ^1^ Digital Health, Technological Innovation Hub (InovaHC); Hospital das Clínicas HCFMUSP, Faculdade de Medicina, Universidade de São Paulo, São Paulo, São Paulo, Brazil.; ^2^ Pulmonary Division, Heart Institute (InCor); Hospital das Clínicas HCFMUSP, Faculdade de Medicina, Universidade de São Paulo, São Paulo, São Paulo, Brazil.; ^3^ Department of Obstetrics and Gynecology, Hospital das Clínicas HCFMUSP, Faculdade de Medicina, Universidade de São Paulo, São Paulo, São Paulo, Brazil.

**Keywords:** COVID-19, respiratory failure, intensive care, telemedicine, digital health

## Abstract

In this scientific report, we aimed to describe the implementation and expansion of a Tele-Intensive Care Unit (Tele-ICU) program in Brazil, highlighting the pillars of success, improvements, and perspectives. Tele-ICU program emerged during the COVID-19 pandemic at the Hospital das Clínicas da Faculdade de Medicina da Universidade de São Paulo (HCFMUSP), focusing on clinical case discussions and training of health practitioners in public hospitals of the state of São Paulo in Brazil, to support health care professionals for treating COVID-19 patients. The success of implementing this initiative endorsed the project expansion to other five hospitals from different macroregions of the country, leading to the Tele-ICU-Brazil. These projects assisted 40 hospitals, allowing more than 11,500 teleinterconsultations (exchange of medical information between health care professionals using a licensed online platform) and training more than 14,800 health care professionals, reducing mortality and length of hospitalized patients. A segment in telehealth for the obstetrics health care was implemented after detecting these were a susceptible group of patients to COVID-19 severity. As a perspective, this segment will be expanded to 27 hospitals in the country. The Tele-ICU projects reported here were the largest digital health ICU programs ever established in Brazilian National Health System until know. Their results were unprecedented and proved to be crucial for supporting health care professionals nationwide during the COVID-19 pandemic and guide future initiatives in digital health in Brazil's National Health System.

## Introduction

Tele-Intensive Care Unit (Tele-ICU) has shown, for a range of medical expertise, a useful practice to provide better health care management of critically ill patients, incorporate new medical technologies, agile patient's treatment, and improve their outcomes.^1^ In this context, Lilly et al. concluded that Tele-ICU interventions increased the adherence to ICU best practices, encouraged the use of performance data, and overall, reduced the mortality rate.^2^ In another study, Khunlertkit and Carayon verified a reduction on ICU length of stay and mortality; they also concluded that Tele-ICU can support medication management and safety, as well as reducing the risks related to ICU health care practices. The authors highlight the contribution of Tele-ICU in improving family's perception in regard to patient care.^3^

The COVID-19 pandemic demanded digital health approaches for coping with the critical demands in the health systems worldwide. During the pandemic, the Hospital das Clínicas da Faculdade de Medicina da Universidade de São Paulo (HCFMUSP) was a reference hospital in the fight against COVID-19 in Brazil, mainly because it's health care professionals developed expertise in the treatment of patients with severe respiratory symptoms by the application of mechanical ventilation (MV) strategies.

Thus, the HCFMUSP specialists developed a validated protocol to COVID-19 acute respiratory distress syndrome (ARDS), which was promptly adopted in the public health care system of the state of São Paulo in Brazil. The Tele-ICU program emerged at the HCFMUSP in a project partnership with the state of São Paulo Health Department (SES). It focused on clinical case discussions and training of health care professionals in innovative medical practices, in public hospitals of the São Paulo state, to support the COVID-19 patients' treatment.^4^

**FIG. 1.**
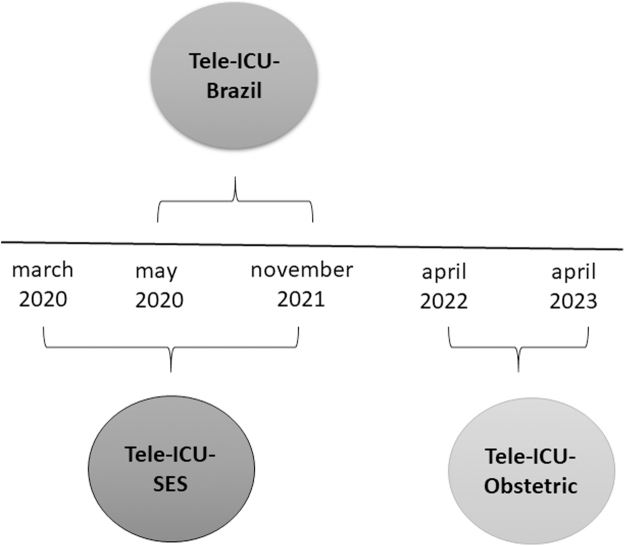
Tele-ICU program timeline. Tele-ICU, Tele-Intensive Care Unit.

The success of Tele-ICU-SES was observed by the World Bank, which, in partnership with the Brazilian Ministry of Health (BMH), funded the expansion of the project to hospitals in different macroregions of the country, promoting the new Tele-ICU-Brazil project. These projects have always been guided by three pillars: training, teleinterconsultations, and prospection of Key Performance Indicators (KPIs) as well as their management. Teleinterconsultation has been defined as a virtual secure space where doctors and other health care professionals discuss clinical cases remotely.^5^ During virtual meetings, they share their expertise in handling protocols for treatment of patients from primary to tertiary care. The Tele-ICU program timeline can be seen in Figure 1.

Thus, the aim of this study was to describe the implementation and expansion of a Tele-ICU program in Brazil, highlighting the pillars of the successful implementation, the relevant improvements, unfolded projects, and perspectives so far.

## Tele-ICU-SES: The First Project in the State of São Paulo

The Tele-ICU-SES project lasted for 20 months, between March 2020 and November 2021 (Fig. 1). In the training segment, we aimed to standardize the knowledge about the care of patients with COVID-19, therefore promoting the autonomy of the participant hospitals. The training activities were carried out by video and online tutorials available at official portals. The protocol for the treatment of COVID-19 patients was made available through free online platforms on the website of HCFMUSP School of Continuing Education and SES-SP,^6^ aiming for a wider dissemination of the training. In addition, a free access mobile application was developed to make learning activities more dynamic, interactive, and accessible. The COVID19VM App had been downloaded more than 33,000 times until August 2022.

Two asynchronous training sessions were made available *on demand*: one in 2020, named “Care for patients with COVID-19,” which included 30 h of workload, and another one in 2021, named “Respiratory Assistance in COVID-19” with 15 h. The target audience consisted of doctors (17.7%), respiratory therapists (33.5%), nurses (33.4%), and nursing assistants (15.4%). Overall, 37 health care institutions participated in the project, with a total of 14,443 trained professionals. We observed a high level of engagement and satisfaction of the participants by measuring the Net Promoter Score (NPS). For these training sessions, we obtained an NPS of 94.

The NPS is a measure of customer loyalty developed by private companies and introduced by Reichheld FF in 2003 for a *Harvard Business Review* article.^7^ The principle of the metric is the belief that customers are promoters, extremely satisfied users (80–100), or detractors (0–60), extremely dissatisfied users. These measurements are used as indicators for qualifying a service according to customers' point of view.

As a complementary activity of the asynchronous training, synchronous sessions were also scheduled by videoconference. These sessions involved clinical case discussions, which emphasized theoretical content as a problem-solving support activity. A total of 8 synchronous sessions were performed during the project, with a total of 676 participants (11% of doctors, 49% of respiratory therapists, 24% of nurses, 14% nursing assistants, and 2% of other health care professionals) and achieving an NPS of 85.

In detail, the teleinterconsultation involved the discussion of selected clinical cases that were in progress at the participating hospital. The possibility of exchanging knowledge allowed a better health care practice for the patient and facilitated the use of new treatment approaches. The teleinterconsultation service achieved an NPS of 82. The Research Electronic Data Capture (REDCap) platform was used to store patients' clinical information, ensuring data confidentiality. The KPIs were established before the beginning of the project; those indexes were monitored weekly to evaluate the progress of the project and also to propose tools for mitigation of problems or to assure goals achievement.

**FIG. 2.**
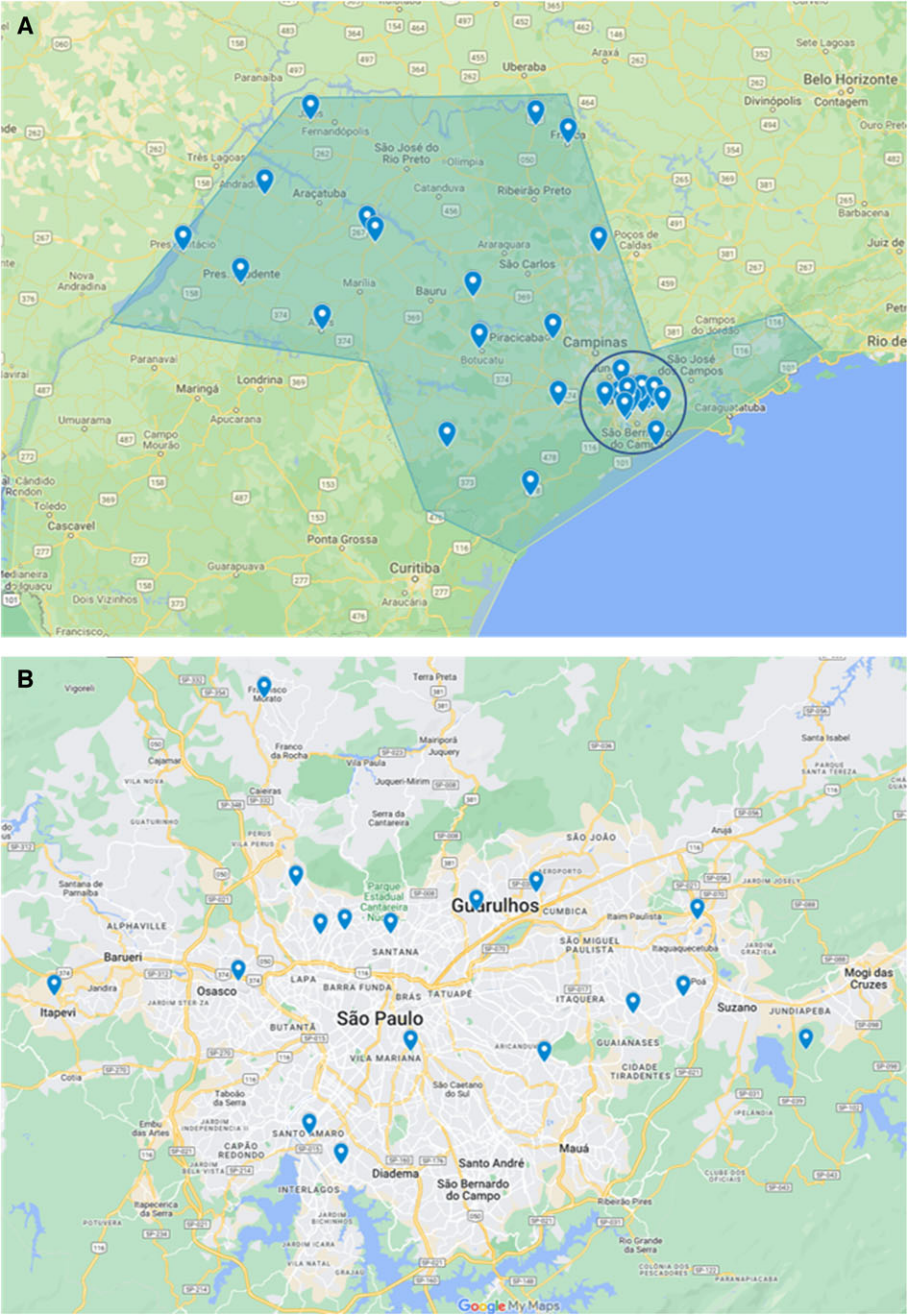
**(A)** Map of the state of São Paulo, Brazil, showing the distribution of the 34 hospitals assisted by the Tele-ICU-SES project across the states (*blue markers*). It is possible to visualize (highlighted in the *circle*) that most of these hospitals were concentrated in the metropolitan region of São Paulo city, the capital of the state of São Paulo. **(B)** Map of the metropolitan region of São Paulo city, showing the distribution of the 17 hospitals assisted by the Tele-ICU-SES project located in the metropolitan region of São Paulo city (*blue markers*). SES, São Paulo Health Department. Reference: Google. 2022. State of São Paulo with the Tele-ICU-SES Network in blue markings: Google Maps. https://www.google.com/maps/d/u/0/edit?mid=1azST-pKkD8AhtI2th_-Jk6zu9s2p8Qc&usp=sharing

The Tele-ICU-SES project assisted 34 hospitals located in 25 cities of the state of São Paulo-Brazil (Fig. 2). During this project, 11,823 teleinterconsultations were performed, counting more than 1700 clinical case discussions (often more than 1 discussion per case), and 14,000 health care professionals were trained. We observed on the Tele-ICU-SES that ICU mortality was 63.6%, lower than that registered in Brazil, for COVID-19 patients (80%).^8^ In the context of the clinical cases, target of the teleinterconsultations (Table 1), some patients maintained clinical characteristics similar to those previously reported in the preliminary assessment as described by Macedo et al.,^4^ such as an average age of 60 years, 58% male, Simplified Acute Physiology Score 3 (SAPS3) of 53, including comorbidities including arterial hypertension 49%, obesity 42%, and diabetes mellitus 32%.

**Table 1.** Data obtained from the projects of Tele-Intensive Care Unit, including patients' profile, severity, and outcome

Variable	Tele-ICU-SES	Tele-ICU-Brazil
Hospitals, *n*	34	5
Patients, *n*	1698	82
Age (years), median (IQR) [*n*]	60 (42–66) [1698]	47 (34–65) [82]
Male sex, % (*n*/*N*)	58 (985/1698)	40 (33/82)
Pregnancy/postpartum patients, % (*n*/*N*)^a^	13 (42/329)	26 (16/62)
COVID-19 positive, % (*n*/*N*)	75.7 (1286/1698)	78 (64/82)
SAPS3, median (IQR) [*n*]	53 (44–62) [434]	53 (44–68) [53]
MV, % (*n*/*N*)	89 (658/739)	90 (74/82)
ICU mortality, % (*n*/*N*)	63.6 (1079/1698)	61 (46/75)
Mortality in pregnancy/postpartum, % (*n*/*N*)	24 (10/42)	31.2 (5/16)

Values are presented as median (IQR), *n* or % (*n*/*N*).

^a^ Considering only the female patients.

ICU, intensive care unit; IQR, interquartile range; MV, mechanical ventilation; SAPS3, Simplified Acute Physiology Score 3; SES, São Paulo Health Department.

Also, 89% of patients were under MV (above 64% reported in the database of the Brazilian ICUs network)^8^ with a PaO_2_/FIO_2_ ratio of 140, all suggesting for moderate-to-severe ARDS. It was interesting that one of the participating hospitals of the Tele-ICU-SES project had a high demand of COVID-19 case discussions in the obstetric sector (74.2%, 23/31 patients), which agreed with worldwide observations on the susceptibility of this population.

## Tele-ICU Expansion to Another Regions of Brazil

Brazil has a large territory, including several regions of difficult access and with huge socioeconomic disparities. In addition, these regions lack health care infrastructure and specialized professionals. In this context, the health care demands caused by the pandemic worsened the quality of the services provided by the public health system in these areas.^9^ Thus, the results of Tele-ICU-SES motivated our team to expand the project to other regions of the country and to create the Tele-ICU-Brazil project in partnership with the BMH. This project lasted for 7 months, between May 2021 and November 2021 (Fig. 1), and was implemented in five hospitals based on the regions with the greatest need for specialized support in COVID-19 assistance.

**FIG. 3.**
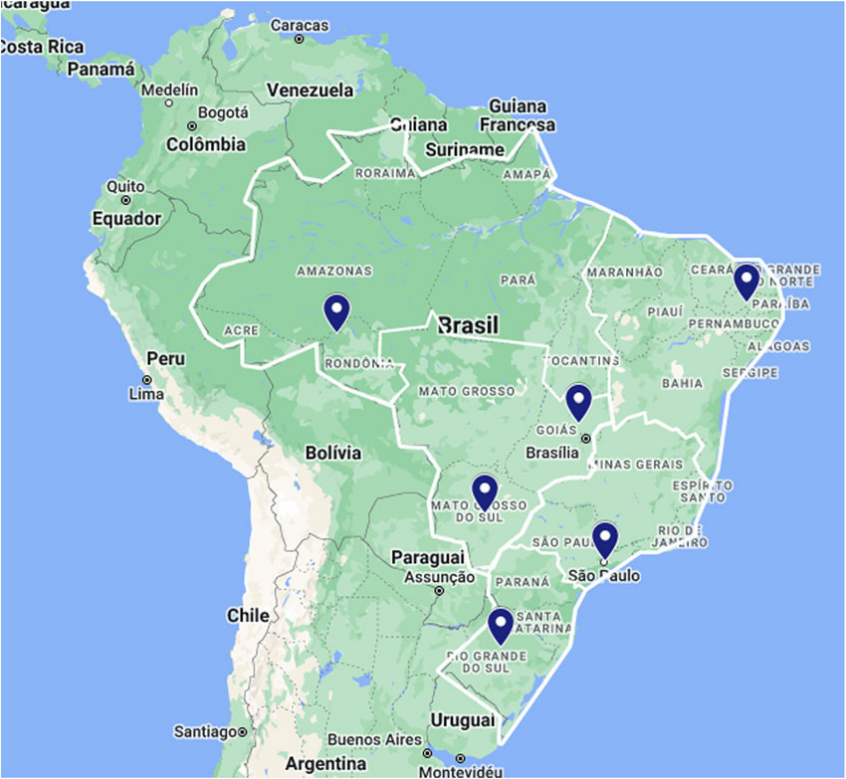
Map of Brazil, showing the distribution of the five hospitals assisted at Tele-ICU-Brazil (*blue markers*), one in each macroregion of the country. Reference: Google. 2022. Brazil with the Tele-ICU Network in blue markings: Google Maps. https://www.google.com/maps/d/u/0/edit?mid=1azST-pKkD8AhtI2th_-Jk6zu9s2p8Qc&usp=sharing

These hospitals were localized in the five macroregions of the country (Fig. 3). Tele-ICU-Brazil maintained the scope and the three pillars of Tele-ICU-SES project. In addition, due to the obstetric cases demand observed in the Tele-ICU-SES, we considered relevant to include a specific attendance to this public in the Tele-ICU-Brazil.

The asynchronous training offered at Tele-ICU-Brazil was entitled “Management of Critical Patients with COVID-19,” and it accomplished four online courses specific to each professional category (10% of doctors, 31% of respiratory therapists, 41% of nurses, and 17% of nursing assistants). The asynchronous training had a total workload of 15 h, a total of 233 professionals enrolled, a completion rate of 20%, and an NPS of 98. There was also a synchronous training, accomplishing 35 events performed during the project progress, involving a total of 735 participants (29% of doctors, 37% of respiratory therapists, 18% of nurses, 14% nursing assistants, and 2% of other health care professionals) and achieving an NPS of 96.

During the Tele-ICU-Brazil project, 82 patients were followed up (Table 1), 936 clinical case discussions were held, with an average of 2.4 discussions per teleinterconsultation. The overall ICU mortality was 61% and 68% among patients in invasive mechanical ventilation (IMV). However, as previously observed in the Tele-ICU-SES project, we could verify an important reduction in these rates during the project progress.

Among the 82 followed patients, we noticed that 26% of the cases (*n* = 16) were obstetric, 12 of whom were pregnant (75%) and 4 were postpartum women (25%). Among them, 14 received MV (87.5%), which is considered a high rate when compared with the national average (87.5% vs. 43%, considering ICU patients).^10^ Among the 12 patients included during pregnancy, 8 were discharged from the hospital (66.6%), 3 patients died (25%), and 1 patient was transferred to the quaternary service (8.3%). Among the four patients who were included in the clinical case discussions during the postpartum period, the mortality rate was 50%. The mortality of monitored obstetric cases was 31.2%.

## Tele-ICU for Assessing High-Risk Obstetric Cases: An Opportunity for the Program Expansion

Even before the pandemic, the maternal mortality was high in Brazil, totaling a rate of 55.3 deaths per 100,000 live births in 2019.^11^ Data from the National Obstetric Observatory showed that the maternal mortality in 2021 varied from 22% to 86% among the states of Brazil, which is considered high, especially because they were mostly young patients with low incidence of comorbidities.^12^ Thus, Brazil has adopted an objective to reduce the maternal mortality until 2030, aiming to diminish to 30 deaths per 100,000 live births.^13^ This measure increased the Brazilian public health awareness to improve the maternal health care.

**FIG. 4.**
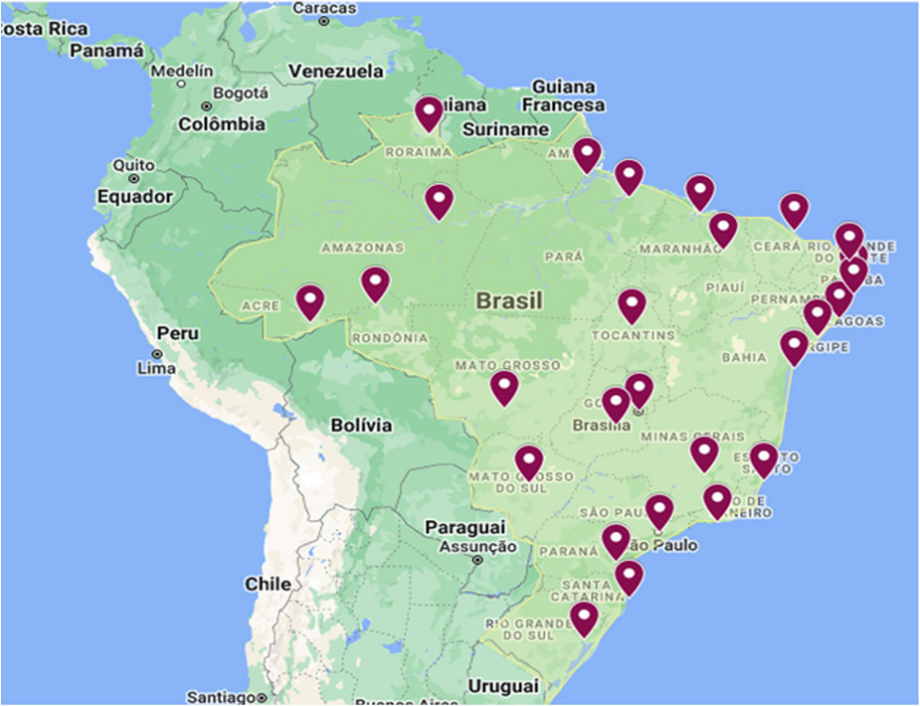
Map of Brazil, showing the distribution of the 27 hospitals that are expected to be assisted by the Tele-ICU-Obstetric project, 1 in each state of the country (*red markers*). Reference: Google. 2022. Brazil with the Tele-ICU Network in red markings: Google Maps https://www.google.com/maps/d/u/0/edit?mid=1azST-pKkD8AhtI2th_-Jk6zu9s2p8Qc&usp=sharing

Looking at our obstetric data (Table 1), the Tele-ICU-Brazil program has already pointed to the necessity to follow-up COVID-19 obstetric cases. Thus, it became evident that Tele-ICU projects, with the aim of qualifying the health care services in obstetrics, would be necessary even in a nonpandemic context. Therefore, in this context, the Tele-ICU-Obstetric project was created in partnership with the BMH. This is an ongoing project that expanded from the Tele-ICU program, and started in April 2022 (Fig. 1), and aims to include 27 hospitals distributed across the Brazilian territory (Fig. 4).

The Tele-ICU-Obstetric project will embrace a training program to any health care professional working on ICU and Obstetrics, and it will first focus in the management of pregnant/postpartum women with obstetric diseases related to maternal mortality, and also expanding to general ICU health care management.

Considering that we intended to disseminate and standardize the health care practices across the Brazilian ICU and Obstetrics working groups, an asynchronous training was developed, and it was entitled “Teletraining in ICU for high-risk obstetric” and counting with 30 h of training sessions. The *on-demand* asynchronous training will be supported by synchronous sessions, and on-site training. Thus, we expect to accomplish not only the hospital's training needs but also cover important aspects listed by our experienced and specialized professionals in intensive care and obstetrics. The on-site training will occur in the designated participant hospitals and will assemble health care professionals with different expertise.

In addition, to survey the effectiveness of the teletraining proposed for the Tele-ICU-Obstetric, and track the project progress, we monitor a set of KPIs. In detail, we chose to evaluate in a week basis and during the whole project the number of teleinterconsultations and clinical cases discussed, the absenteeism rate, and also the number of pregnant women, of postpartum women, abortions, births, fetal deaths, neonatal deaths, and the registered SAPS3 of each patient.

## Conclusions and Perspectives

The experience obtained during the Tele-ICU-SES project and the upscale for the Tele-ICU-Brazil project enabled the training of several health care professionals in Brazil, as well as the remote clinical follow-up of critically ill patients admitted to the ICU during the COVID-19 pandemic. The results have shown that this project improved the health care treatments throughout the country, and it was reflected by the great acceptance of health care professionals who engaged in the online training and in teleinterconsultations as highlighted by the high NPS obtained in the appreciation assessment. Its expansion to the public health care system was unprecedented, and it has been considered the first digital health care ICU program largely implemented in the Brazil's National Health System.

The projects have shown to be extremely important and feasible during the COVID-19 pandemic, not only encouraging the creation of other Tele-ICU-related projects but also expanding the teleconsulting and the offering of specialized treatment to inaccessible and economic deprived regions of the country during and after the pandemic period.

In regard to the challenging aspects for the translation of these institutional initiatives into government continuous programs for the public health system of Brazil, we highlight the difficulty to promote the hospitals adhesion to the project. For example, the critical infrastructure demands for digital health projects implementation in several regions of the country excluded the possibility to increase the number of participating hospitals and limited the project's expansion. We noticed that the project adhesion was also linked to the need of increasing the perception of the participating hospitals to its relevance for the national public health system. In addition, keep participant hospitals engaged in the projects for more than 12 months represented another challenge. Thus, we suggest the 12 months as a feasible period for the implementation of assistance projects.

On the future perspectives, we believe that the development of a dedicated digital health federal program will help to create and develop local health care specialized hubs and leaders with a broad geographical distribution to act as knowledge dissemination centers. The perspectives of the HCFMUSP, in regard of future Tele-ICU projects, aim to fully implement the use of remote technology to improve ICU teleconsulting services in the public health system, and also to include telemonitoring as a new important pillar of our future projects.

In this task, the HCFMUSP aims to promote interoperability among the different health care systems, to integrate the patients' information in a dynamic, and analytical platform, that could be accessed remotely, during the teleinterconsultations. We believe that monitoring patient's health data remotely will enable a faster and improved diagnostic tool, allowing the real-time verification of patient's physiological parameters at the bedside, helping to guide the clinical discussions and treatment approaches. Our perspective is to pave the way for the development of other teleconsulting programs in ICU, stimulating the use of technologies that can facilitate and optimize the health care service.

## Abbreviations Used

ARDS: acute respiratory distress syndrome
BMH: Brazilian Ministry of Health
IQR: interquartile range
KPI: Key performance indicators
MV: mechanical ventilation
NPS: Net Promoter Score
SAPS3: Simplified Acute Physiology Score 3
SES: São Paulo Health Department
Tele-ICU: Tele-Intensive Care Unit
